# AMN Congress 2022 – Report of the panel on TBI specifics and pragmatic solutions for improvement in low- and middle-income countries

**DOI:** 10.25122/jml-2022-1014

**Published:** 2022-07

**Authors:** Alexandra-Mihaela Gherman, Dafin Fior Muresanu, Andreea Strilciuc

**Affiliations:** 1. RoNeuro Institute for Neurological Research and Diagnostic, Cluj-Napoca, Romania; 2. Department of Neuroscience, Iuliu Hatieganu University of Medicine and Pharmacy, Cluj-Napoca, Romania

The 4^th^ and last panel of the AMN 19^th^ Congress, which took place on Day 2, May 13, had as proposed theme “TBI in low- and middle-income countries – what are the specifics, what are the pragmatic solutions for improvement” on which the below panelists:

- Felix Brehar (Romania);
- Volodymyr Golyk (Ukraine);
- Ignacio Previgliano (Argentina);
- Tarek Lotfy (Egypt);

alongside Prof. Dafin Muresanu invitees:

- Andriy Huk (Ukraine);
- Johannes Vester (Germany);
- Nicole von Steinbüchel (Germany);
- Stefan Strilciuc (Romania);
- Razvan Chereches (Romania);
- Irina Vlad (Romania);
- Iulia Vadan (Romania);
- Constantin Radu (Romania).

provided insightful and action-oriented analyses offering a glimpse of the future steps to be taken in the field of TBI.

Prof. Dafin Muresanu used PRESENT (Patient REgistry – Short Essential NeuroTrauma), designed to bring more clarity to the flow of TBI patients and the data to be collected, as an example of a project developed in a middle-income country like Romania. Furthermore, Johannes Vester called upon condensing the essence of the new scientific research by bringing together all pieces of the puzzle for a deeper understanding of the complex setting of trauma, as well as of the complexity of therapy options at different points in time and different groups of patients, from mild to severe. Thus, a multidisciplinary approach such as the one embraced by AMN also means discussing health care issues.

The entire dialogue of the panelists and invitees was constructed upon the country data sheets presented by some specialists, the first one belonging to the Romanian representative, Felix Brehar. Important data regarding the present status of the Traumatic Brain Injury (TBI) care system were shared, starting with epidemiological references such as incidence, similarity with an average incidence in EU countries, and mortality rates. The etiology of TBI is split between traffic accidents (50.5%), falls (29.7%), aggression (17.7%), loss of consciousness (0.8%), and other unspecified conditions (1.3%). At the same time, the management of TBI, especially mild cases, follows a route that starts from the emergency unit, followed by a CT scan, the neurosurgical department, and followed by actions undertaken either by the neurologist or the family physician, with a 60 days follow-up after discharge at home. A similar route is present in the case of severe TBI, with admission into the Intensive Care Unit (ICU) after the Computerized Tomography (CT) is performed, and a rehabilitation program undertaken after the release from the neurosurgical department. Several weak points of the status of TBI care are also presented, such as the lack of CT scans in all small hospitals in the country, of biomarkers in TBI management (S100B protein [1] has been proven to rule out negative CT scans in mild TBIs), incomplete implementation of standard procedures regarding neurotrauma, scarce communication between neurotrauma centres across the country, good rehabilitation infrastructure present only in a few centres, and difficulty in achieving complete follow-up visits. The solutions proposed include 4 objectives to be met during a 3-year time span, *i.e*.:

- By 2023 – Implementation of neurotrauma programs by extending the existing program (that covers only cranioplasty) or implementing new programs;
- By 2023 – Accessing EU grants by submitting more project proposals dedicated to TBI basic and clinical research to the Horizon EU program;
- By 2024 – Setting up a TBI national registry employing a dedicated server information system that collects all the information regarding hospitalized patients using the diagnosis-relateg group (DRG) system;
- By 2025 – Setting up 3 rehabilitation centres part of county hospitals using EU funds and 3 rehabilitation centres part of country hospitals using national funds.

With respect to future directions in TBI research, special attention was given to the biomarkers approach. More precisely, as CT scans are used excessively in the emergency departments for mild TBIs, the use of S100B protein – blood biomarker screening test (detectable in serum within minutes from injury, can be assayed using an automated device in 18 minutes, has a sensitivity of 95–100% *etc*.) to rule out negative CT scans becomes a necessity. Should an S100B analysis be negative, the patient could be discharged with oral and written information to be considered as per the minimal brain injury diagnosed.

The second country sheet, based on data assessed from the central database of Hospital Fernandez in Argentina, was presented by Ignacio Previgliano. He used a timespan of 20 years to analyse his data, *i.e*., 2001 to 2019 and 2021. While the incidence of head injury by age or mechanism of the lesion did not change during this time span, enormous changes occurred regarding the head injury incidence among over 10.5 million inhabitants (from 322 injuries in 2001 to 109 in 2019 and 47 in 2021, respectively), most probably due to the pandemic. As a result, the incidence in the emergency ward and the severity of head injury changed accordingly. For example, there were only 7 cases of severe head injury in 2021, while the assessed mortality following a severe head injury decreased from 35% in 2001 to 25% in 2019 and further down to 23.50% in 2021.

Ignacio Previgliano concluded that a possible explanation for the decrease of all the assessed parameters could be the improvement of care processes in the emergency room and intensive care unit (increased use of POCUS – point-of-care ultrasound to diagnose problems and RECUSS protocol – ultrasound guided resuscitation of the patient).

Final information was provided regarding the coverage of the National Traumatic Database in Argentina, namely:

- 1 hospital (the main hospital) in Buenos Aires city;
- 22 hospitals in Buenos Aires state;
- 4/5 public hospitals in different states.

As a final remark, I. Previgliano added that ICU monitoring devices (bought by the Ministry of Health) are available in all city hospitals and this enhances major advances to be made in managing severe head trauma [2].

Moving on with the presentations, Prof. Dafin Muresanu invited the colleagues from Ukraine to offer their perspectives on the panel topic [3]. Volodymyr Golyk used a pre-/post- start of war dichotomy to point to the (pre-) existence of the medical guarantees in health care, which covered 3 packages for rehabilitation, out of which 1 was designated for neurorehabilitation – TBI included. After the war started, TBI was included in the overall blast trauma (combined with complex limb injuries, burns, amputations, *etc*.). Currently, the program of medical guarantees is declared the ex-territorial principle of delivery and rehabilitation so that everybody can receive this service anywhere. Volodymyr Golyk underlines that, during this period, there is a need for a pathway, with support from the Ministry of Health and international organizations, to help identify non-destroyed facilities that could be used as rehabilitation centres, but also to enhance capacity building with respect to staff, equipment, and overall system rebuilt.

In addition to what his colleague stated, Andriy Huk points to the need for a good IT infrastructure to work with digitalised applications. Having previously tested the RES-Q (Registry of Stroke Care Quality) application, where an average of 15 minutes were necessary to work with the patient, Andriy Huk stated that the clear need-to-know data for TBI is an immediate necessity and, without the implementation of registries, the quality of health care could not be influenced in terms of improvements brought in.

The problem of documenting official numbers in TBI is also present in Egypt, as the country is divided into high-income and low-income regions. Tarek Lotfy points to the lack of specialised centres for head injuries and treatment of TBI and the scarcity of intradepartmental connectivity and communication where these specialisations exist [4].

As a public health specialist, Razvan Chereches highlights some conclusions from an analysis he undertook in 2019 regarding the number of published articles connected to TBI, namely that high-income countries have the highest number of articles published, with the highest impact [5]. Given that in those countries there are registries available, valuable literature can also be generated that could be further used to increase visibility and attract funds. On the other hand, for low- and middle-income countries, support could be provided for accessing funds/grants but also for transferring know-how that could be adjusted to the environment in question [6].

With Prof. Dafin Muresanu asking what would be the best meeting point between the efforts of AMN and the actions of the governmental structure regarding the issue of registries, Stefan Strilciuc, governmental expert, indicated that, as Romania is shifting from a middle-income to a high-income country (income based on GDP), the level of care is close to that of a low-income country, which also impacts research to a great extent. Concerning national registries, the major problem would be the lack of capacity to implement projects at the central level, so, from his perspective, the solution would come from the academic field/professional societies. To support his statement, Stefan Strilciuc used PRESENT, a project of the AMN where the simplicity and brevity of the tool is key to gathering compliance from the physicians, but also RES-Q – a registry for quality of care in stroke that benefited from a lot of support from the industry. In other words, the solution is to come up with something simple but able to bridge the need for information and mirror the clinical process.

Prof. Dafin Muresanu emphasised the statements of Stefan Strilciuc, highlighting the good direction of PRESENT and RES-Q, with the latter being upgraded to close the loop with the rehabilitation part and the former offering a clear, simple, time-saving model for doctors and generating useful information.

Constantin Radu, also an expert currently training in public health and health economics, underlined the importance of mapping out the patient's pathways and identifying the best interventions, not only in terms of efficiency and safety, but also in terms of cost-effectiveness so that a set of interventions are put together, and offer the best outcome for the patient.

Moreover, as PRESENT enables data collection from physicians in a timely and effective manner, it should become the rule for data gathering, optimizing patient pathways, and identifying the most cost-effective interventions.

Moreover, Iulia Vadan highlighted the accessibility of PRESENT to be used by patients and the rapidity in providing status information and tracking patients. Irina Vlad underlined that this registry was tailored to the needs of TBI and, as such, it covers the multidisciplinarity of TBI and the patient's pathway from the ER to discharge (the average time needed for completing the registry is 7.3 minutes). Shortly, it will also function as a tool for hospitals without an electronic database of patients, *i.e*., via means of artificial intelligence (AI), to gather data from existing databases of patients.

Considering all of the above, the entire development of the Congress, and in reference to the Neurotrauma Treatment Simulation Center (NTSC) – VIENNA upcoming event that would also provide hard evidence to contribute to a better understanding of the field problems related to TBI, Prof. Dafin Muresanu concluded that the Academy for Multidisciplinary Traumatology is a mature organization, with stable pillars and clear projects and task forces at work for developing future projects. Nicole von Steinbüchel added that the AMN has all the requisites and motivation to move forward, integrate younger people, and also work on ameliorating patient care.

The president of AMN, Johannes Vester, closed the panel, the session, and the Congress with his statement that there is only ONE HUMAN BEING (*i.e*., the patient) that counts and this way of thinking should be enhanced across academic borders.
